# The Elephants in the Room: Sex, HIV, and LGBT Populations in MENA. Intersectionality in Lebanon: Comment on "Improving the Quality and Quantity of HIV Data in the Middle East and North Africa: Key Challenges and Ways Forward"

**DOI:** 10.15171/ijhpm.2016.149

**Published:** 2016-12-04

**Authors:** Rachel L. Kaplan, Cynthia El Khoury

**Affiliations:** ^1^University of California, San Francisco, CA, USA.; ^2^Arab Foundation for Freedoms and Equality, Beirut, Lebanon.

**Keywords:** HIV, Data, Middle East and North Africa (MENA), LGBT, Political Context

## Abstract

In response to this insightful editorial, we wish to provide commentary that seeks to highlight recent successes and illuminate the often unspoken hurdles at the intersections of culture, politics, and taboo. We focus on sexual transmission and draw examples from Lebanon, where the pursuit of data in quality and quantity is teaching us lessons about the way forward and where we are experiencing many of the challenges referenced in the editorial such as discrepancies between national statistics and rates derived via research as well as the impact of protracted political conflict and displacement. Two important points were raised in the editorial about HIV in Middle East and North Africa (MENA) that we would like to expand further: (1) The epidemic is largely driven by drug-related and sexual behavior among key populations; and (2) Several key populations continue to be criminalized and excluded from surveillance programs.

In their recent editorial, authors Karamouzian, Madani, Doroudi, and Haghdoost provided an important examination of why the quality and quantity of HIV data in the Middle East and North Africa (MENA) has been limited.^1^ Karamouzian et al successfully described the recent body of literature that addresses HIV rates in the region and identified potential reasons that the data are lacking among both the general population and key populations in MENA countries. Indeed, although MENA is one of the only regions throughout the world that has not been able to curb rising HIV incidence rates, the research, data, and programming within the region does not match the obvious need for focused effort and attention. The editorial succinctly explores potential reasons that might explain this imbalance of evidence of need versus available data and offers useful future strategies.

“There is a wide perception that the socio-cultural and political sensitivities associated with HIV research in the MENA as well as structural-level stigma, may restrict access to HIV-related data and create barriers in conducting HIV research in the region.”^1^

The above quotation from the editorial represents a central challenge faced by those who are interested or engaged in HIV research in MENA. Because the epidemic is largely driven by sexual and drug-related behavior, frank discussions and financial commitments at the national level prove challenging. The cultural landscape in MENA is such that HIV testing is often enshrouded in fear and shame by the test taker and suspicion and stigma by others. That HIV is transmitted through behavior that transgresses social, moral, and legal code in many countries further complicates the goal of accurate data.

The authors identified key populations at urgent risk of HIV: people who inject drugs (PWID), men who have sex with men (MSM), female sex workers (FSWs) and their sexual and injecting partners. They also noted other important populations requiring priority: foreign migrant workers, military personnel, refugees, street children, and internally displaced individuals. We would like to remind readers of two important points regarding key populations: (1) Transgender populations, with particular priority for trans feminine people including transgender women, are among the most-at-risk individuals^2^ and are often still missing in our discussions about key populations or erroneously conflated with MSM^3,4^; and (2) Many individuals navigate HIV risk at the intersections of multiple risk categories. For example, some transgender women engage in sex work. Some MSM are refugees. These intersecting identities represent urgent need requiring prioritized action and cultural understanding of how this intersectionality impacts health risk.

“Several key populations (eg, FSWs, MSM) continue to be criminalized and are excluded from surveillance programs due to several socio-political sensitivities.”^1^

The above quotation from the editorial speaks to the intersection of human rights and public and global health. This statement could very well capture the crux of HIV data limitations and must be acknowledged first and foremost. Given the fact that discussions about sex, particularly outside marriage, are taboo in addition to HIV-related stigma that permeates society, HIV data collection among the general population is incredibly challenging. On top of an already difficult task, engaging key populations in research introduces a wide range of seemingly insurmountable hurdles. Is it ethical to request participation from populations who by their very identity experience discrimination, violence, assault, and arrest? How can we protect participants who are most-at-risk and vulnerable? Before police engagement recommendations can be made, we must ask ourselves about the history of police involvement with key populations whose lives and health we aim to improve. Involvement of security forces may endanger key populations given the current climate in some countries. In fact, police forces have historically treated key populations with bullying, violence, and assault in contexts throughout the world including MENA.

Understanding the context in which people living with HIV/AIDS (PLWHA) and key populations live is essential for navigating future directions. PLWHA face stigma and discrimination, for example, in employment discrimination and deportation within the region. PLWHA who are also members of key populations likely experience compounding and intersecting stigma.^5^ Further, HIV testing has been used to intimidate key populations. During a raid of a “gay-friendly” venue in 2014 in Beirut, those arrested were forced to submit to HIV testing; results were used coercively to control detainees’ testimonies.^6^ Until human rights violations are addressed, and rights recognized and ensured, progress will be limited. When we ask countries within MENA to fund HIV research, we must examine the social, moral, and legal status of those individuals whose lives would be directly impacted.

For those of us researchers who are based outside MENA, many of us have privilege that can be used to bring attention to the health needs of the region. We have educational privilege, border crossing ease, and infrastructure and financial access that prioritize information exchange. Given the challenges that countries in the region face to fund “politically unsafe” HIV research, cross-region partnerships are key. However, without connection, access, or entrée with and in communities of key populations, progress can be difficult. Because of histories fraught with discrimination, violence, and even death, many members of LGBT (lesbian, gay, bisexual, and transgender) populations globally can be wary of developing trust with outsiders; in contexts that do not provide protections, rapport and engagement can be understandably slow. Researchers and/or organizations with pre-formed agendas that fail to identify or incorporate key populations’ priorities miss opportunities for real engagement. There is a need for community-led research that traditional funding mechanisms do not afford. Further, in efforts to resist the exoticization of Arab culture, and in particular Arab gay bodies, some individuals and groups oppose collaboration. Thus, it is the very nature of shared identities that both promote and discourage collaboration. LGBT researchers outside MENA might be able to develop trust and rapport with counterparts within the region, but long-term involvement may prove impossible due to the identity-related lack of safety when in-country. Similarly, LGBT researchers within the region may not be safe “coming out” about the connection they have to their work.

## Additional Hurdles and Negotiating Risks

Because some MENA countries choose not to allocate funds and/or lack the political will to commit to HIV data collection and programming, international funders and collaborators are essential. Although international investments in HIV research in MENA have improved (eg, In 2008, the funding opportunity announcement from the US National Institutes of Health [NIH] for HIV research in MENA: http://grants.nih.gov/grants/guide/pa-files/PAR-08-153.html), political relations between MENA and other regions pose incredible logistical challenges when securing funding and setting up contracts with organizations in MENA. Further, because of the perceived or actual risk of travel to some MENA countries and government-issued travel warnings, researchers outside MENA may face obstacles when trying to obtain permissions for staff and students to participate in research activities, thereby limiting efficiency, collaboration, training, and sustainability. It is possible that these challenges will worsen in the United States, for example, given the impact of Islamophobia and anti-Middle Eastern sentiment, the 2016 election climate, and the lack of transparency about foreign policy from presidential candidates.

Partnerships between individuals and organizations in MENA and other regions, by definition, are impacted by the complexities of geo-political relations and positionality. Activists and young researchers are often scrutinized and/or judged by their communities when collaborating with Western researchers. The second author has been told by fellow activists on several instances that she is “collaborating with the enemy” and that she is “indirectly informing foreign intelligence on our communities” because she is working on research that is funded by the NIH. Some organizations in MENA do not accept financial support from American funders for political reasons. Likewise, some stipulations of American federal funding can make it difficult if not impossible for researchers to accept monetary support for scholarship. For example, the first author, when pursuing doctoral research support in 2009 had to decline such funding because of a personal relationship at the time with a Lebanese citizen. Accepting the funding would have required willingness to answer detailed questions about personal relationships. Such questions would have put the Lebanese citizen at risk because of LGBT identity. Further, unlike counterparts doing international HIV research who are not LGBT and are, therefore, able to travel or relocate to research sites, LGBT researchers must consider the safety and legal implications of not only themselves, but also partners/spouses and children. Similarly, LGBT researchers in MENA risk exposure via engagement in the work and memberships or collaborations with LGBT organizations based both within and outside MENA.

## Successes

Despite these challenges, there are some exciting new developments, particularly in Lebanon, that address some of the most-at-risk populations mentioned above. We now have quality data from Lebanon about condom use, HIV testing, and unprotected anal sex among male refugees who have sex with men from two recent Lebanese-led publications.^7,8^ We also have quality data from Lebanon about suicide risk, HIV prevalence, HIV risk factors and categories, and sexual health threats among trans feminine individuals.^3,5,9,10^ Efforts to address health disparities among trans feminine people in Lebanon are currently underway.

## Strategies

In an effort to reach members of LGBT communities who are most-at-risk for HIV, true allies, who are willing to be trained for working with LGBT groups, are key. Many researchers within the region who are not LGBT are potential allies and can work toward health equity with LGBT partners. Similarly, many researchers outside the region who are not LGBT can move about freely without fear of violence and discrimination. Partnerships across identities will prove strongest and most sustainable. Another strategy to learn about best practice for HIV data collection in MENA would be to survey all researchers who have obtained quality HIV data about engaging key populations, leveraging funds, and sustaining efforts over time. As noted in the editorial, size estimation studies are needed and challenging; the region suffers from changing populations due to political conflict, forced migration and displacement, migration within and across regions for employment opportunities, and punitive laws. Additionally, culturally appropriate intervention strategies are necessary; such strategies must not only be culturally relevant to diverse MENA contexts, but also to the identities, cultures, and languages of the targeted groups and populations.

## Ethical issues

Not applicable.

## Competing interests

This article was prepared by Rachel L. Kaplan and Cynthia El Khoury in their personal capacity. The opinions expressed in this article are the authors’ own and do not reflect the view of the National Institutes of Health (Bethesda, MD, USA), the United States government, Arab Foundation for Freedoms and Equality (Beirut, Lebanon), San Francisco State University (San Francisco, CA, USA), or the University of California, San Francisco (CA, USA).

## Authors’ contributions

Concepts, design, and manuscript editing: RLK, CEK; Literature search and manuscript drafting: RLK; Both of the authors made substantial suggestions for the revisions of the manuscript and approved the final submitted version of the paper.

## Authors’ affiliations

^1^University of California, San Francisco, CA, USA. ^2^Arab Foundation for Freedoms and Equality, Beirut, Lebanon.
